# The Growth and Protein Expression of Inflammatory Bowel Disease-Associated *Campylobacter concisus* Is Affected by the Derivatives of the Food Additive Fumaric Acid

**DOI:** 10.3389/fmicb.2018.00896

**Published:** 2018-05-17

**Authors:** Rena Ma, Fang Liu, Soe F. Yap, Hoyul Lee, Rupert W. Leong, Stephen M. Riordan, Michael C. Grimm, Li Zhang

**Affiliations:** ^1^The School of Biotechnology and Biomolecular Sciences, University of New South Wales, Sydney, NSW, Australia; ^2^Concord Hospital, University of New South Wales, Sydney, NSW, Australia; ^3^Gastrointestinal and Liver Unit, The Prince of Wales Hospital, Sydney, NSW, Australia; ^4^St George and Sutherland Clinical School, University of New South Wales, Sydney, NSW, Australia

**Keywords:** *Campylobacter concisus*, sodium fumarate, enteric bacteria, food additives, inflammatory bowel disease

## Abstract

Inflammatory bowel diseases (IBD) are chronic inflammatory conditions of the gastrointestinal tract with multifactorial etiology. Both dietary factors and the microbe *Campylobacter concisus* have been found to be associated with the condition. The current study examined the effects of sodium fumarate, a neutralized product of the food additives fumaric acid and monosodium fumarate when in the intestinal environment, on the growth of *C. concisus* to determine the effects of these food additives on IBD-associated bacterial species. Through culture methods and quantification, it was found that neutralized fumaric acid, neutralized monosodium fumarate, and sodium fumarate increased the growth of *C. concisus*, with the greatest increase in growth at a concentration of 0.4%. Further examination of 50 *C. concisus* strains on media with added sodium fumarate showed that greatest growth was also achieved at a concentration of 0.4%. At a concentration of 2% sodium fumarate, all strains examined displayed less growth in comparison with those cultured on media without sodium fumarate. Using mass spectrometry, multiple *C. concisus* proteins showed significant differential expression when cultured on media with and without 0.4% sodium fumarate. The findings presented suggest that patients with IBD should consider avoiding excessive consumption of foods with fumaric acid or its sodium salts, and that the addition of 0.4% sodium fumarate alone to media may assist in the isolation of *C. concisus* from clinical samples.

## Introduction

Inflammatory bowel disease (IBD) refers to the inflammatory disorders affecting the gastrointestinal tract, including Crohn’s disease (CD) and ulcerative colitis (UC) (Cosnes et al., 2011). The symptoms of CD and UC are very similar, though the pathology of each differs, allowing for diagnosis. Inflammation in CD may occur in any part of the gastrointestinal tract with a discontinuous pattern, while the inflammation in UC is continuous with diffuse and superficial lesions (Cosnes et al., 2011). Currently, the etiology of IBD is unknown, and several factors are suggested to contribute to its pathogenesis, such as host microbiota and diet (Sartor, 2006). *Campylobacter concisus* is an oral bacterium that has been linked to IBD. There was a significantly higher prevalence of *C. concisus* detected in intestinal biopsies of patients with IBD as opposed to healthy controls (Zhang et al., 2010; Mahendran et al., 2011; Mukhopadhya et al., 2011; Kirk et al., 2016). Studies have also shown that some oral *C. concisus* strains possess the ability to damage the intestinal epithelial barrier, suggesting that translocation of these strains to the lower gastrointestinal tract may increase the risk of the individual developing IBD (Nielsen et al., 2011; Ismail et al., 2012; Zhang, 2015; Mahendran et al., 2016). *C. concisus* strains are comprised of two genomospecies (Aabenhus et al., 2005; Engberg et al., 2005; Kalischuk and Inglis, 2011; Miller et al., 2012; On et al., 2013; Mahendran et al., 2015; Chung et al., 2016; Nielsen et al., 2016) and recently we have found that *C. concisus* genomospecies 2 strains were better adapted to the gastrointestinal environment (Wang et al., 2017).

Dietary factors have long been associated with a range of chronic illnesses. In IBD, there was a positive correlation of high intake of carbohydrates and fats to IBD incidence (Sakamoto et al., 2005). Non-Western countries historically have a low incidence and prevalence of IBD, but are showing an increasing incidence and prevalence of IBD coinciding with industrialization and westernization (Drewnowski and Popkin, 1997; Cosnes et al., 2011). The societal changes in these non-Western countries shift the diet toward high fat, high sugar, and processed foods, which are characteristic of Western diets, suggesting that dietary factors are likely to contribute to IBD incidence (Pingali, 2007). In murine models, it was shown that a high fat and high sugar diet changes the gut microbiota composition and promoted the colonization of IBD-associated adherent-invasive *Escherichia coli* (Martinez-Medina et al., 2014). With processed foods becoming increasingly consumed due to low cost and convenience achieved by food additives and industrial ingredients (Monteiro et al., 2013), their effects on IBD-related bacterial species require investigation.

Fumaric acid (E297) is a widely used acidity regulator (Smith and Hong-Shum, 2011), with its sodium salts, monosodium fumarate and sodium fumarate (E365) used less commonly for the same purposes. While the use of sodium fumarate as a food additive has been discontinued in Europe, it is allowed in Australia and New Zealand. There is no limit on the amount of fumaric acid used in both United States and Australia (CFR 172.350 in the United States, Standard 1.3.1 in Australia), while in the European Union a maximum of 4000 mg/L has been suggested with voluntary enforcement [Regulation (EC) no 1333/2008]. The neutralization of fumaric acid (C_4_H_4_O_4_) via contact with the naturally occurring sodium bicarbonate in the intestinal tract can produce both monosodium fumarate and sodium fumarate. At less acidic pH, fumaric acid dissociates to form monosodium fumarate (NaC_4_H_3_O_4_) and then sodium fumarate (Na_2_C_4_H_2_O_4_) at almost neutral pH (Roa Engel et al., 2013).

Previous studies utilized the combined supplementation of sodium formate and sodium fumarate into culture media to isolate *C. concisus* (Tanner et al., 1981; Macuch and Tanner, 2000). This suggests that sodium fumarate can increase *C. concisus* yield. Therefore, we hypothesize that sodium fumarate, the neutralized product of the food additive fumaric acid, affects the growth of *C. concisus* and protein expression. This hypothesis was examined in this study.

We found that sodium fumarate supplementation into culture media alone affected the growth of *C. concisus* and some other enteric bacterial species. In addition, sodium fumarate altered the protein expression in *C. concisus*.

## Materials and Methods

### Bacterial Strains Used in This Study

A total of 50 *C. concisus* strains were examined in this study. Of these, 49 strains were isolated from previous studies with ethics approval granted by the Ethics Committees of the University of New South Wales and the South East Sydney Area Health Service, Australia [HREC09237/SESIAHS 09/078, HREC08335/SESIAHS(CHN)07/48, HREC06233/SESAHS(ES)06/164, HREC09237/SESIAHS09/078, and HREC08335/SESIAHS(CHN)07/48] (Zhang et al., 2010; Mahendran et al., 2011). *C. concisus* strain 13826 was purchased from the American Type Culture Collection. The details of the *C. concisus* strains used in this study and genomospecies, if known (Mahendran et al., 2015; Chung et al., 2016; Wang et al., 2017), are shown in **Table 1**. Additionally, four other enteric species obtained from the American Type Culture collection were included in this study as representatives of other species in the enteric environment (Bachmann, 1972; Gregory et al., 1978; Moschetti et al., 1998): *Bacteroides fragilis* ATCC 25285, *Bacteroides vulgatus* ATCC 8482, *Enterococcus faecalis* ATCC 19433, and *E. coli*.

**Table 1:** Bacterial strains used in this study.

Strain ID	Health status	Source	Genomospecies
P10CDO-S1	CD	Saliva	1
P10CDO-S2	CD	Saliva	1
P11CDO-S1	CD	Saliva	1
P19CDO-S1	CD	Saliva	1
P20CDO-S4	CD	Saliva	1
P25CDO-S3	CD	Saliva	1
P27CDO-S2	CD	Saliva	1
P26UCO-S2	UC	Saliva	1
P3UCB1	UC	Intestinal biopsy	1
P3UCLW1	UC	Fecal sample	1
P3UCO1	UC	Saliva	1
H1O1	Healthy	Saliva	1
H10O-S1	Healthy	Saliva	1
H12O-S1	Healthy	Saliva	1
H15O-S1	Healthy	Saliva	1
H17O-S1	Healthy	Saliva	1
H21O-S3	Healthy	Saliva	1
H24O-S1	Healthy	Saliva	1
H25O-S1	Healthy	Saliva	1
H26O-S1	Healthy	Saliva	1
H26O-S1	Healthy	Saliva	1
H27O-S1	Healthy	Saliva	1
H28O-S1	Healthy	Saliva	1
P1CDO2	CD	Saliva	2
P1CDO3	CD	Saliva	2
P2CDO4	CD	Saliva	2
P6CDO1	CD	Saliva	2
P12CDO-S1	CD	Saliva	2
P18CDO-S1	CD	Saliva	2
P20CDO-S2	CD	Saliva	2
P20CDO-S3	CD	Saliva	2
P21CDO-S1	CD	Saliva	2
P21CDO-S2	CD	Saliva	2
P24CDO-S2	CD	Saliva	2
P24CDO-S3	CD	Saliva	2
P27CDO-S1	CD	Saliva	2
P26UCO-S1	UC	Saliva	2
P7UCO-S2	UC	Saliva	2
P8UCO1	UC	Saliva	2
P13UCO-S3	UC	Saliva	2
P16UCO-S1	UC	Saliva	2
P16UCO-S2	UC	Saliva	2
H3O1	Healthy	Saliva	2
H7O-S1	Healthy	Saliva	2
H9O-S2	Healthy	Saliva	2
H11O-S1	Healthy	Saliva	2
H14O-S1	Healthy	Saliva	2
H22O-S1	Healthy	Saliva	2
H29O-S1	Healthy	Saliva	2
13826	Gastroenteritis	Fecal sample	2


*The 50 strains of *C. concisus* were previously isolated from our studies. The letters P and H under Strain ID indicate a patient with IBD or a healthy control, respectively, and the following number is unique for separate individuals. UC, CD, and H under Health status indicate whether the strain was isolated from a patient with ulcerative colitis, Crohn’s disease, or a healthy control, respectively. Genomospecies status known from previous studies is listed (Mahendran et al., 2015; Chung et al., 2016; Liu et al., 2018).*

### Examination of the Effects of Sodium Fumarate and Sodium Formate on *C. concisus* Growth

We first examined whether individual supplementation of fumarate is comparable to supplementation with both sodium fumarate (Sigma-Aldrich, St. Louis, MO, United States) and sodium formate (Sigma-Aldrich), five strains of *C. concisus* were randomly chosen. These strains were first cultured on horse blood agar (HBA, Oxoid, Hampshire, United Kingdom) plates consisting of 6% defibrinated horse blood (Oxoid) in anaerobic conditions with 5% hydrogen as previously described (Lee et al., 2014). Cultures with an optical density of 0.1 at a wavelength of 595 nm (OD_595_ 0.1) were prepared (Ma et al., 2015), and 5 μL was inoculated onto HBA, HBA with 0.2% sodium formate (HBA^form^), HBA^fum0.4^, and HBA plates with both 0.2% sodium formate and 0.4% sodium fumarate (HBA^fum+form^) and streaked for single colonies. After incubation, all plates were examined for colony size differences by visual inspection and plate photographs were obtained through a Samsung HZ30W digital camera attached to the stereomicroscope at a magnification of 20×.

The effects of sodium fumarate and sodium formate on the growth of *C. concisus* were also quantified through CFU determination of the randomly chosen *C. concisus* strain, P26UCO-S2. P26UCO-S2 was cultured first cultured on HBA as described above before inoculation onto HBA, HBA^fum0.4^, HBA^form^, and HBA^fum+form^ in triplicates and incubated for 72 h. After incubation, cultures were collected and serially diluted for bacterial enumeration using the drop plate method as previously described (Ma et al., 2015). Experiments were repeated three times.

### Quantitative Assessment of the Effect of Neutralized Fumaric Acid and Neutralized Monosodium Salt on *C. concisus*

The pH of H_2_O solutions containing 0.05, 0.2, 0.4, 1, and 2% fumaric acid, monosodium fumarate, or disodium fumarate was measured with a pH meter (Eutech pH700, Eutech Instrument, Singapore). The solutions of fumaric acid and monosodium fumarate were acidic and it was previously shown that low pH abolishes the viability of *C. concisus* (Ma et al., 2015). However, the ingested fumaric acid and monosodium fumarate would be neutralized in the lower gastrointestinal tract. Therefore, to determine the possible effects of the neutralized ingested fumaric acid and sodium monofumarate, neutralized solutions of fumaric acid and monosodium fumarate were examined.

Solutions of 20% (w/v) fumaric acid (Sigma-Aldrich) and monosodium fumarate (Nippon Shokubai, Osaka, Japan) were neutralized with sodium bicarbonate (Sigma-Aldrich) and used as the stock solution. *C. concisus* strains P26UCO-S1 and P2CDO4 were chosen randomly to be examined. Cultures of OD_595_ 0.1 were prepared as described in the Section “Examination of the Effects of Sodium Fumarate and Sodium Formate on *C. concisus* Growth” and 5 μL was inoculated onto HBA and HBA supplemented with 0, 0.05, 0.2, and 0.4% fumaric acid adjusted to pH 7 (HBA^FA0.05^, HBA^FA0.2^, and HBA^FA0.4^) or monosodium fumarate adjusted to pH 7 (HBA^MF0.05^, HBA^MF0.2^, and HBA^MF0.4^) in triplicate and spread in a radial pattern with a sterile hockey stick. Plates were incubated for 72 h and colony-forming units (CFU) were quantified as described in the Section “Examination of the Effects of Sodium Fumarate and Sodium Formate on *C. concisus* Growth.” Experiments were conducted three times.

### *C. concisus* Cultivation for the Observation of Colony Size Differences in the Presence of Sodium Fumarate

As both neutralized monosodium fumarate and neutralized fumaric acid increased growth of *C. concisus*, further experiments were performed with the neutralized final product, sodium fumarate. All 50 *C. concisus* strains were cultured and suspensions of OD_595_ 0.1 were prepared as described in the Section “Examination of the Effects of Sodium Fumarate and Sodium Formate on *C. concisus* Growth.” From these bacterial suspensions, 5 μL was streaked for single colonies onto a HBA plate and HBA plates supplemented with 0.05% (HBA^fum0.05^), 0.4% (HBA^fum0.4^), 1% (HBA^fum1^), and 2% (HBA^fum2^) sodium fumarate in triplicates and the plates were incubated for 72 h. After incubation, plates were examined and photographed as described in the Section “Examination of the Effects of Sodium Fumarate and Sodium Formate on *C. concisus* Growth.”

### Quantitative Assessment of the Effects of Different Concentrations of Sodium Fumarate on the Growth of *C. concisus* and Other Enteric Bacterial

From the 50 strains of *C. concisus* examined for morphological colony changes above, 7 strains were randomly selected for quantitative assessment of growth in the presence of different concentrations of sodium fumarate, including P2CDO4, P11CDO-S1, and P20CDO-S3 from patients with CD, P26UCO-S1 from a patient with UC, H12O-S1, and H17O-S1 from healthy controls and *C. concisus* strain 13826. Furthermore, the growth of *B. fragilis*, *B. vulgatus*, *E. faecalis*, and *E. coli* were also quantified.

Four different concentrations of sodium fumarate were supplemented into HBA plates, including the HBA^fum0.05^, HBA^fum0.4^, HBA^fum1^, and HBA^fum2^ used previously in this Section. *C. concisus* strains were first cultured on HBA plates as described in the Section “Examination of the Effects of Sodium Fumarate and Sodium Formate on *C. concisus* Growth.” Bacterial cells were harvested, diluted to an OD_595_ of 0.1 and 5 μL of the suspension was spread plated onto HBA plates and HBA^fum0.05^, HBA^fum0.4^, HBA^fum1^, and HBA^fum2^ plates in triplicate and incubated for 72 h. After incubation, cultures were collected and serially diluted for bacterial enumeration using the drop plate method as previously described (Ma et al., 2015). Experiments were repeated three times.

*Bacteroides fragilis*, *B. vulgatus*, *E. faecalis*, and *E. coli* were cultured as previously described (Liu et al., 2017). Bacterial pellets were then collected, diluted and cultured as described for all *C. concisus* strains above. Due to their faster growth rates, these species were incubated for 24 h instead of 48 h (Liu et al., 2017).

### Quantitative Assessment of the Growth of *C. concisus* When Cultured With and Without Sodium Fumarate Over 96 h

As the previous experiment found that a concentration of 0.4% sodium fumarate was preferable for the growth of most strains, this concentration was then used to examine the growth of *C. concisus* over a period of 94 h to examine the effect of sodium fumarate on growth rate. Three strains were randomly chosen to observe their growth when cultured on media with and without sodium fumarate.

All three strains were cultured on HBA plates as previously described and bacteria were collected. To prevent bacterial oversaturation of the agar plates, a lower OD_595_ of bacteria were used. Each strain was diluted to an OD_595_ of 0.025 and 5 μL of each were spread plated onto HBA and HBA^fum0.4^ in triplicates for enumeration at 0, 12, 24, 48, 72, and 96 h. After incubation for the respective amount of time, plates were removed from incubation and the bacteria were collected and quantified as previously described (Ma et al., 2015). Additionally, wet mounts of each strain were also prepared at each time point to observe the morphology of *C. concisus*.

### Quantification of the Growth of 50 Strains of *C. Concisus* on Media With and Without Sodium Fumarate

Based on the results of the Section “Quantitative Assessment of the Growth of *C. concisus* When Cultured With and Without Sodium Fumarate Over 96 h,” the biggest effect of 0.4% sodium fumarate on *C. concisus* could be observed at 24 h; consequently, this condition was utilized to quantify the growth of all *C. concisus* strains in this study. All strains of *C. concisus* were cultured as per the Section “Examination of the Effects of Sodium Fumarate and Sodium Formate on *C. concisus* Growth” and diluted to an OD_595_ of 0.025. From the bacterial suspensions, 5 μL were spread plated onto HBA and HBA^Fum0.4^. Plates were incubated for 24 h before bacteria were harvest and serially diluted for enumeration as described previously in the Section “Examination of the Effects of Sodium Fumarate and Sodium Formate on *C. concisus* Growth.”

### Analysis of the Proteins of *C. concisus* 13826 When Cultured With and Without Sodium Fumarate

From the *C. concisus* strains examined, strain 13826 (accession number: NC_009802) was chosen as the representative strain for protein analysis to examine changes in protein profile when cultured with and without sodium fumarate as its genome is readily accessible from the National Center for Biotechnology Information. First, *C. concisus* strain 13826 was cultured on HBA for 48 h. The bacterial cells were then collected, prepared and cultured on HBA and HBA^fum0.4^ as described in the Section “Examination of the Effects of Sodium Fumarate and Sodium Formate on *C. concisus* Growth.”

After incubation, bacteria were harvested and washed according to the Section “Examination of the Effects of Sodium Fumarate and Sodium Formate on *C. concisus* Growth.” Whole cell lysate proteins were prepared by three cycles of rapid freezing and thawing. Protein concentration was determined via the bicinchoninic acid method according to manufacturer’s instructions (Pierce, Rockford, IL, United States) and 20 μg of proteins were subjected to SDS–PAGE. Mass spectrometry was performed on proteins as previously described (Ismail et al., 2012; Luu et al., 2017). Experiments were repeated three times.

Peak lists were generated using mascot daemon/extract_msn (Matrix Science, London, United Kingdom), with default parameters, and all MS/MS spectra were searched against the NCBIprot_3_11_15 database (68653660 entries, specificity selected for bacteria) using MASCOT (version 2.5.1, Matrix Science, London, United Kingdom). Data search parameters were as follows: precursor tolerance was 4 ppm, and product ion tolerances were ± 0.4 Da; oxidation of methionine, carbamidomethyl alkylation of cysteine, and propionamide on cysteine was specified as a variable modification, enzyme specificity was trypsin and one missed cleavage was possible.

Scaffold Q+ (v.4.7.3, Proteome software, Portland, OR, United States) was used to validate peptide and protein identities (Searle, 2010). Peptide identifications were accepted if they could be established at greater than 95% probability by the Protein Prophetalgorithm (Nesvizhskii et al., 2003). Proteins were accepted if they could be established at greater than 99% probability and contained at least 2 identified peptides. Only proteins present in two or more biological replicates were included. Normalized spectral counts in Scaffold provided an estimation of relative protein quantification. Protein function was derived from the eggNOG database (Muller et al., 2010).

### Statistical Analysis

The comparisons of CFU and total protein variation of *C. concisus* strains cultured on the varying media with the CFU on the control HBA plates were obtained through *t*-tests. To compare relative abundances of proteins, *t-*tests were also performed with multiple test correction using the Benjamini–Hochberg method (Benjamini and Hochberg, 1995). A *p*-value of less than 0.05 was considered significant.

## Results

### Comparison of *C. concisus* Growth on HBA Plates Supplemented With Sodium Fumarate Alone With the Combined Supplementation of Sodium Fumarate and Sodium Formate

To investigate the effects of sodium fumarate and sodium formate on colony size, the growth of five *C. concisus* strains were examined. In all five strains, there was a visible decrease in colony size on HBA^form^ in comparison with HBA, while all *C. concisus* on HBA^fum0.4^ showed an increased colony size. On HBA^fum+form^, there was no visible difference in colony sizes compared to HBA plates.

**FIGURE 1:**
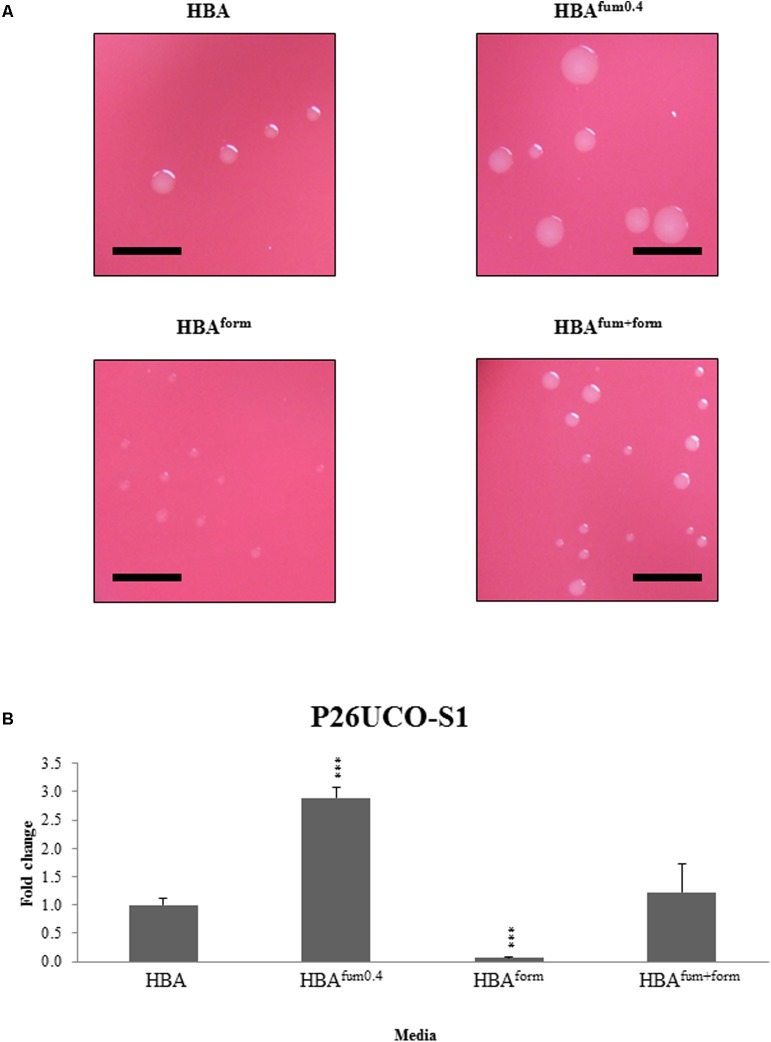
Quantitative analysis of sodium fumarate and sodium formate on the growth of *C. concisus*. **(A)** Colonies of representative *C. concisus* strain P26UCO-S2. A total of five *C. concisus* strains were streaked onto HBA plates, HBA plates with 0.4% sodium fumarate (HBA^fum0.4^), HBA with 0.2% sodium formate (HBA^form^), and HBA with both sodium fumarate and sodium formate (HBA^fum+form^). After incubation the colonies were observed under a stereomicroscope and photographs were captured at 20× magnification. Scale bars represent 5 mm. Of the five strains examined, all showed increased colony size when cultured on HBA^fum0.4^ and decreased colony size on HBA^form^. All strains showed no visible difference in colony size when cultured on HBA^fum+form^. **(B)** The fold changes in CFU of *C. concisus* strain P26UCO-S2 cultured on HBA, HBA^fum0.4^, HBA^form^, and HBA^fum+form^ is shown. Fold changes were calculated relative to the CFU of the same strain cultured on plates without fumarate or formate from the mean of quadruplicate counts on each media. ^∗∗∗^ indicates *p* < 0.001.

To further investigate the effects of the supplements, the CFU of strain P26UCO-S2 was quantified. The CFU of P26UCO-S2 when supplemented with 0.4% sodium fumarate and 0.2% sodium formate was not significantly different to HBA. When cultured on HBA^fum0.4^, the CFU increased significantly (*p* < 0.05) in comparison with HBA. On HBA^form^, the CFU showed a significant decrease (*p* < 0.05 in comparison with HBA) (**Figure 1**).

### Measurement of *C. concisus* Growth on HBA Plates Supplemented With Neutralized Fumaric Acid or Neutralized Monosodium Fumarate

**FIGURE 2:**
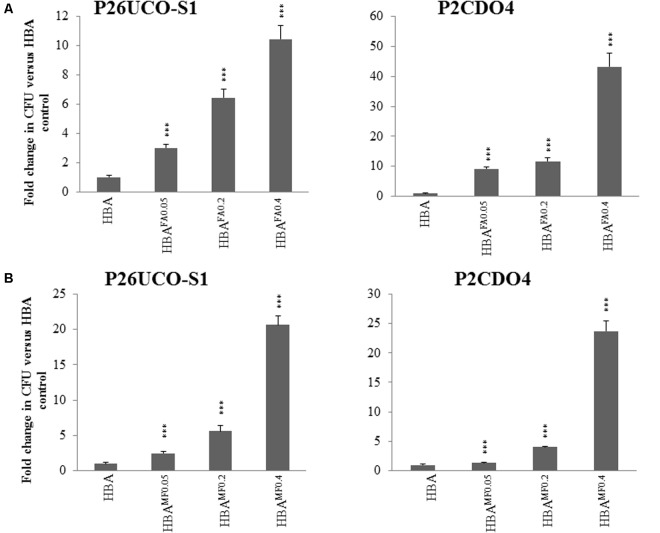
Quantitative analysis of neutralized fumaric acid and neutralized monosodium fumarate on the growth of *C. concisus*. P26UCO-S1 and P2CDO4 are both *C. concisus* strains, the former isolated from a patient with UC and the latter from a patient with CD. Both strains were quantified after growth on HBA plates supplemented with varying concentrations of **(A)** neutralized fumaric acid and **(B)** neutralized monosodium fumarate. Fold changes were calculated relative to the CFU of the same strain cultured on plates without supplementation from the mean of quadruplicate counts on each media. ^∗∗∗^ indicates *p* < 0.001.

The pH of different concentrations of fumaric acid, monosodium fumarate, and sodium fumarate were measured and are shown in **Table 2**. *C. concisus* strains P26UCO-S1 and P2CDO4 representing *C. concisus* genomospecies 1 and 2, respectively, were examined for growth on HBA plates supplemented with neutralized monosodium fumarate or neutralized fumaric acid. Both strains had significantly increased growth on HBA^MF0.05^ compared with HBA (*p* < 0.05), with P26UCO-S1 showing a fold change of 2.5 and P2CDO4 showing a fold change of 1.3. On HBA^MF0.2^, both P26UCO-S1 and P2CDO4 showed a further increase in fold change, with a fold change of 5.6 and 4, respectively. The greatest increase in fold change could be seen on HBA^MF0.4^ for both strains, with P26UCO-S1 increasing to 20.1 times the CFU on HBA and P2CDO4 increasing 23.7 times (**Figure 2**).

**Table 2:** The pH of fumaric acid and its sodium salts at different concentrations.

Concentration of solute	Fumaric acid	Monosodium fumarate	Sodium fumarate
0.05%	2.96	3.81	7.54
0.2%	2.76	3.67	7.22
0.4%	2.55	3.68	7.75
1%	2.5	3.65	7.76
2%	2.48	3.6	7.92

*Fumaric acid, monosodium fumarate, and sodium fumarate solutions were made by suspension in distilled water at concentrations of 0.05, 0.2, 0.4, 1, and 2%.*

When cultured on HBA^FA0.05^, both *C. concisus* strains P26UCO-S1 and P2CDO4 showed a significant increase in CFU compared to on HBA plates, with fold changes of 3 and 9.1, respectively. As the concentration of neutralized fumaric acid increased, the CFU of P26UCO-S1 also increased with fold changes of 6.4 on HBA^FA0.2^ and 10.4 on HBA^FA0.4^. Similarly, for P2CDO4, the fold changes on HBA^FA0.2^ and HBA^FA0.4^ were significantly increased P26UCO-S1 and P2CDO4, at 11.7 and 43.3, respectively.

### Changes in Colony Size on HBA Plates With and Without Sodium Fumarate

**FIGURE 3:**
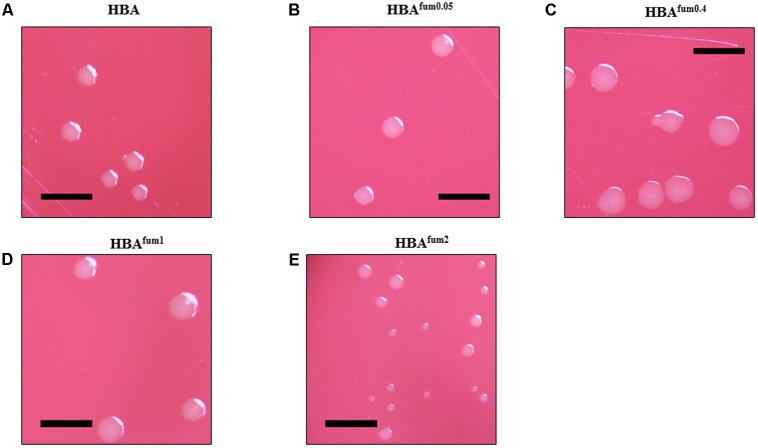
The effects of sodium fumarate on the colony size of *C. concisus*. Colonies of representative *C. concisus* strain H14O-S1 on HBA plates **(A)** without supplementation and **(B–E)** with supplementation of sodium fumarate at 0.05, 0.4, 1, and 2%, respectively. Scale bars represent 5 mm. A total of 50 *C. concisus* strains were streaked onto HBA, HBA^fum0.05^, HBA^fum0.4^, HBA^fum1^, and HBA^fum2^. After incubation, colonies were observed under a stereomicroscope and photographs were captured at 20× magnification.

When cultured on HBA^fum0.05^, 27 of the 50 *C. concisus* strains examined showed an increase in colony size compared to the same strain on HBA. The remaining 23 stains showed no visible change in colony size compared to the same strain on HBA. On HBA^fum0.4^, all strains of *C. concisus* tested showed visibly larger colonies as compared to that of the same strain on HBA, although the colony size increments varied in different strains. When cultured on HBA^fum1^, 45 of 50 strains examined noticeably decreased in colony size as compared to that of the same strain cultured on HBA. From the five remaining strains, four showed no obvious difference in colony size compared to the controls and the final strain was P11CDO-S1, which showed an increase in colony size compared to the control. When the concentration of sodium fumarate was increased to 2%, 6 of the examined strains showed complete inhibition of growth. All of the remaining strains had a clearly smaller colony size in comparison with HBA. Photos of the results of a randomly chosen representative strain, H14O-S1, are shown in **Figure 3**.

### Quantitative Comparison of the Effects of Sodium Fumarate on *C. concisus* Growth

**FIGURE 4:**
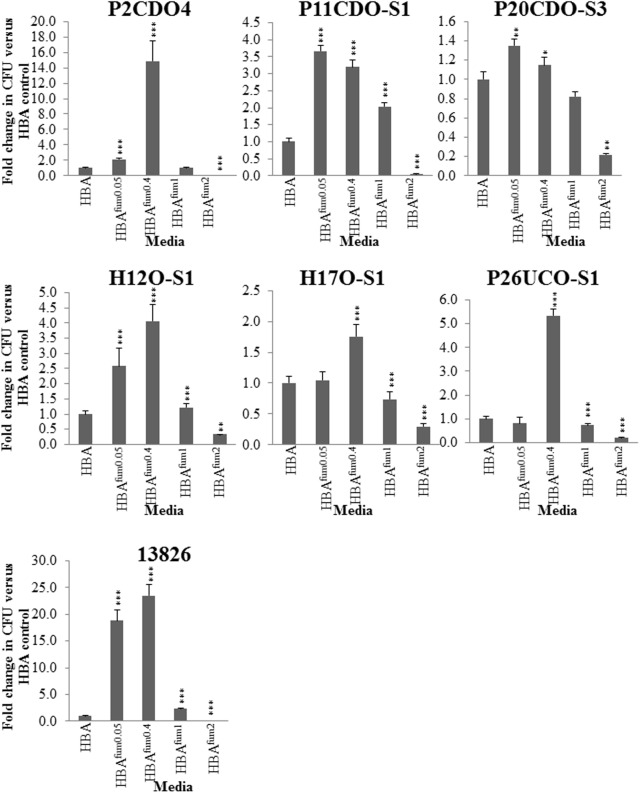
Quantitative analysis of *C. concisus* cultured in the presence of varying concentrations of sodium fumarate. Seven randomly chosen strains of *C. concisus* were grown on HBA plates with varying concentrations of sodium fumarate; the number following Fum indicates the percentage of sodium fumarate in the HBA plate. Each strain was then collected and the CFU were enumerated. Fold changes were calculated relative to the CFU of the same strain cultured on HBA from quadruplicate counts. ^∗^ indicates *p* < 0.05, ^∗∗^ indicates *p* < 0.01, and ^∗∗∗^ indicates *p* < 0.001.

To further characterize the impact of sodium fumarate on the growth of *C. concisus*, the growth of seven strains of *C. concisus* were quantified after culturing on HBA supplemented with varying concentrations of sodium fumarate. When cultured on HBA^fum0.05^, the CFU of five *C. concisus* strains (P2CDO4, P11CDO-S1, P20CDO-S3, H12O-S1, and 13826) were significantly increased compared to that on HBA (**Figure 4**). The CFU of *C. concisus* strain 13826 was increased significantly (*p* < 0.05), with an 18.8-fold change compared to its CFU on HBA plates. The remaining 4 strains had fold changes of between 1.3 and 2.7 on HBA^fum0.05^ compared to the same strain on HBA. All strains of *C. concisus* examined showed a significant increase in CFU (*p* < 0.05) when cultured on HBA^fum0.4^ as compared to HBA, with fold changes of 1.2- to 24.5-fold in CFU when cultured on HBA^fum0.4^ as compared to HBA. When cultured on HBA^fum1^, strains showed a differential response with strains 13826, H12O-S1 and P11CDO-S1 showing an increase of 1.2- and 3.2-fold in CFU, respectively, P26UCO-S1 and H17O-S1 both showing 0.7-fold decrease in CFU, and P2CDO4 and P20CDO-S3 showing no change (*p* > 0.05) in CFU as compared to that on HBA (**Figure 4**). However, when cultured on HBA^fum2^, all examined strains of *C. concisus* consistently showed a decrease in CFU as compared to the same strain cultured without sodium fumarate, with fold changes between 0.05 and 0.3.

### Effects of Sodium Fumarate on the Growth of Other Enteric Species

**FIGURE 5:**
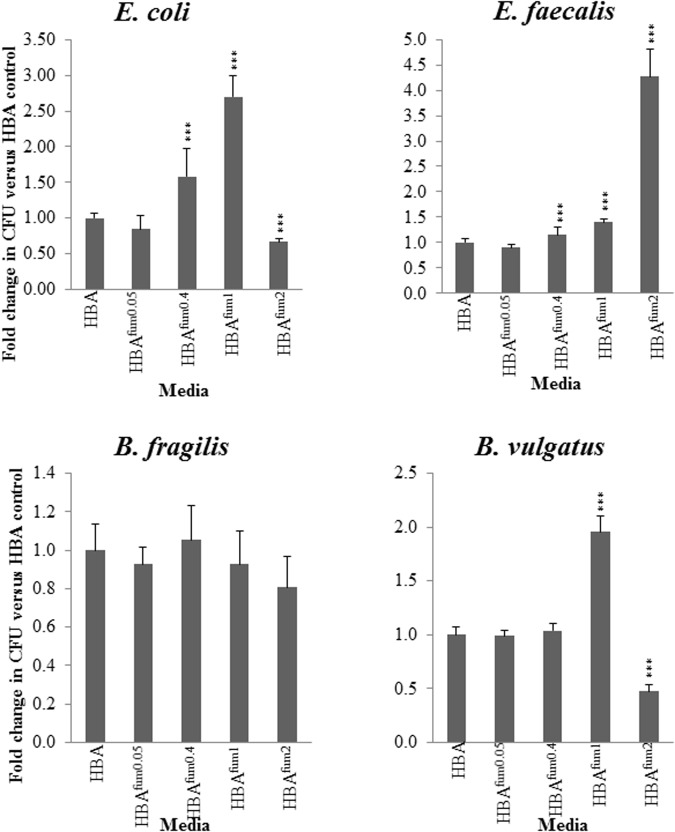
Quantitative effects of varying concentrations of sodium fumarate on enteric bacterial species. *B. fragilis*, *B. vulgatus*, *E. faecalis*, and *E. coli* were chosen as representative bacteria of the enteric environment. Each species was cultured on HBA plates with varying concentrations of sodium fumarate; the number following Fum indicates the percentage of sodium fumarate in the HBA plate. Bacteria were then collected and the CFU were quantified. Fold changes were calculated relative to the CFU of the same species cultured on HBA from quadruplicate counts. ^∗∗∗^ indicates *p* < 0.001.

For all the examined enteric species, there was no significant change between the CFU when cultured on HBA^fum0.05^ as compared to HBA. *E. coli* and *E. faecalis* showed similar results on HBA^fum0.4^ with growth significantly increased (*p* < 0.05) compared to HBA. *B. vulgatus* showed no change in CFU (**Figure 5**). When the concentration of sodium fumarate is increased to 1%, *E. coli*, *E. faecalis*, and *B. vulgatus* showed a significant increase (*p* < 0.05) in CFU. On HBA^fum2^, *E. coli* and *B. vulgatus* displayed a significant decrease in CFU. *E. faecalis* showed a significantly increased (*p* < 0.05) CFU compared to HBA plates.

At all concentrations of sodium fumarate examined, *B. fragilis* showed no significant difference in CFU compared to the CFU on HBA plates (*p* > 0.05).

### Growth Curve of *C. concisus* Cultured on HBA and HBA^Fum0.4^

**FIGURE 6:**
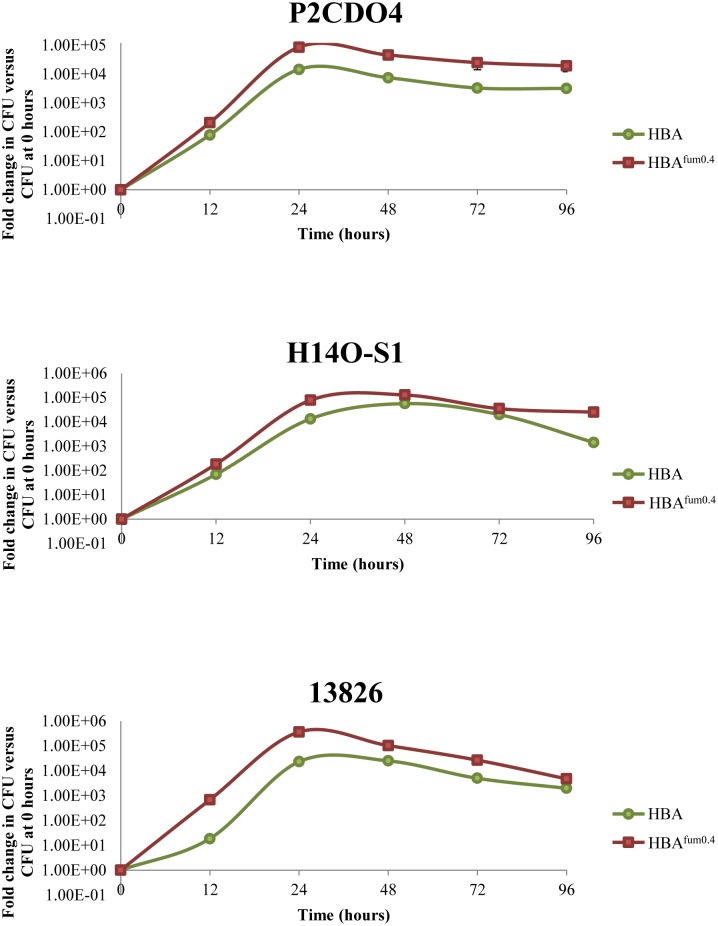
Growth of *C. concisus* strains P2CDO4, H14O-S1, and 13826 cultured on HBA^fum0.4^ and HBA over 96 h. A. Each strain was cultured on HBA and HBA with 0.4% sodium fumarate (HBA^fum0.4^) and the total bacteria were enumerated at 0, 12, 24, 48, 72, and 96 h after incubation. Results are represented as a CFU fold change relative to the CFU at 0 h of incubation from quadruplicate counts.

In the growth curves of all three strains of *C. concisus* examined, marked increase in CFU could be observed when cultured on HBA^fum0.4^ as compared to HBA as early as after 12 h of incubation (**Figure 6**). All three strains maintained a higher overall CFU when cultured on HBA^fum0.4^ as compared to HBA at the same incubation time. While there was an increase in CFU on HBA^fum0.4^, the trend of CFU increase and decrease followed the same pattern as that on HBA in all three strains. From the wet mount preparations, no difference in bacterial morphology could be seen in the same strains on HBA as compared to HBA^fum0.4^ at the same time point.

### Fold Change in Growth of 50 Strains of *C. concisus* When Cultured on Media With and Without Sodium Fumarate

All 50 strains of *C. concisus* examined displayed an increase in growth when cultured on HBA^fum0.4^ as compared to HBA. The increase in CFU varied depending on the strain with the lowest fold change in comparison with HBA on HBA^fum0.4^ being 1.1 ± 0.1 for P20CDO-S3 and the highest fold change being 28.8 ± 1.2 for P10CDO-S2 (**Table 3**). There was no significant difference in the average CFU fold changes from *C. concisus* strains isolated from patient groups (CD nor UC) versus those isolated from healthy controls (*p* > 0.05). No difference in average CFU fold changes were also observed between genomospecies 1 or 2 *C. concisus* (*p* > 0.05).

**Table 3:** CFU fold changes of all 50 strains of *C. concisus* cultured on HBA^fum0.4^ compared to HBA plates.

Strain ID	Genomospecies	Fold change (mean ± SD)
P10CDO-S1	1	12.7 ± 1.3


P10CDO-S2	1	28.8 ± 1.2


P11CDO-S1	1	3.2 ± 0.3


P19CDO-S1	1	1.4 ± 0.0


P20CDO-S4	1	2.3 ± 0.3


P25CDO-S3	1	4.0 ± 0.6


P27CDO-S2	1	16.0 ± 1.9


P26UCO-S2	1	1.6 ± 0.2


P3UCB1	1	17.4 ± 1.9


P3UCLW1	1	3.4 ± 0.1


P3UCO1	1	17.1 ± 2.2


H1O1	1	2.2 ± 0.2


H10O-S1	1	6.5 ± 1.3


H12O-S1	1	1.8 ± 0.4


H15O-S1	1	6.5 ± 1.0


H17O-S1	1	1.3 ± 0.2


H21O-S3	1	20.8 ± 2.0


H24O-S1	1	5.5 ± 0.3


H25O-S1	1	2.1 ± 0.1


H26O-S1	1	9.4 ± 1.5


H26O-S1	1	14.5 ± 0.6


H27O-S1	1	13.6 ± 0.7


H28O-S1	1	22.7 ± 1.4


P1CDO2	2	18.2 ± 11.6


P1CDO3	2	8.5 ± 1.6


P2CDO4	2	5.9 ± 0.6


P6CDO1	2	11.9 ± 2.1


P12CDO-S1	2	10.2 ± 0.8


P18CDO-S1	2	2.0 ± 0.4


P20CDO-S2	2	5.2 ± 0.6


P20CDO-S3	2	1.1 ± 0.1


P21CDO-S1	2	2.8 ± 0.3


P21CDO-S2	2	4.3 ± 0.8


P24CDO-S2	2	7.1 ± 1.4


P24CDO-S3	2	1.4 ± 0.3


P27CDO-S1	2	3.1 ± 0.2


P26UCO-S1	2	14.3 ± 1.6


P7UCO-S2	2	5.8 ± 0.5


P8UCO1	2	1.2 ± 0.1


P13UCO-S3	2	15.8 ± 2.8


P16UCO-S1	2	11.4 ± 1.8


P16UCO-S2	2	10.1 ± 0.8


H3O1	2	6.9 ± 1.2


H7O-S1	2	2.4 ± 0.3


H9O-S2	2	3.6 ± 0.5


H11O-S1	2	18.7 ± 3.4


H14O-S1	2	2.0 ± 0.3


H22O-S1	2	2.2 ± 0.5


H29O-S1	2	10.5 ± 2.3


13826	2	15.5 ± 0.5


*Fold changes were calculated from the CFU of the same strain on HBA^fum0.4^ plates divided by the CFU on HBA plates. Results displayed are the mean fold changes ± the standard deviation (SD). The genomospecies which each strain belongs to is listed beside the Strain ID.*

### Comparison of Proteins Identified in the Whole Cell Lysates of *C. concisus* 13826 Cultured on HBA With and Without Sodium Fumarate

*C. concisus* strain 13826 was used to examine protein expression when cultured on HBA and HBA^fum0.4^. In total, 391 proteins were identified, with 355 being commonly expressed by *C. concisus* cultured on HBA and HBA^fum0.4^. On HBA, there were 9 unique proteins that were not detected when cultured on HBA^fum0.4^ (**Table 4**). When *C. concisus* was cultured on HBA^fum0.4^, there were 27 unique proteins identified (**Table 5**).

**Table 4:** Proteins only identified in *C. concisus* 13826 when cultured on HBA plates without fumarate.

Protein	Accession number	Functional category
Heavy metal translocating P-type ATPase	EAT98303.2	Inorganic ion transport and metabolism
Peptidylprolyl isomerase	WP_002940164.1	Post-translational modification, protein turnover, and chaperones
Peptidase	WP_012000970.1	Amino acid transport and metabolism
Flavocytochrome c	WP_012001109.1	Energy production and conversion
C4-dicarboxylate ABC transporter	WP_012140574.1	Carbohydrate transport and metabolism
Hypothetical protein	WP_012140660.1	Cell wall/membrane/envelope biogenesis
LemA protein	WP_021084760.1	Function unknown
C4-dicarboxylate ABC transporter	WP_048809748.1	Energy production and conversion
UDP-N-acetylmuramate- alanine ligase	WP_048809780.1	Signal transduction mechanisms


**Table 5:** Proteins only identified in *C. concisus* 13826 when cultured on HBA^fum0.4^.

Protein	Accession number	Functional category
Cell division protein FtsA	WP_002940409.1	Cell cycle control, cell division, chromosome partitioning
ATP-dependent protease ATP-binding subunit HslU	WP_002940444.1	Post-translational modification, protein turnover, and chaperones
Twitching motility protein PilT	WP_002941386.1	Cell motility, intracellular trafficking, secretion, and vesicular transport
3-isopropylmalate dehydratase small subunit	WP_002941843.1	Amino acid transport and metabolism
3-isopropylmalate dehydrogenase	WP_002941890.1	Amino acid transport and metabolism
Elongation factor P	WP_004317246.1	Translation, ribosomal structure and biogenesis
Cation ABC transporter substrate-binding protein	WP_004317362.1	Inorganic ion transport and metabolism
DNA-binding response regulator	WP_009294394.1	Signal transduction mechanisms
Amino acid ABC transporter substrate-binding protein	WP_012001169.1	Amino acid transport and metabolism
Competence/damage-inducible domain-containing protein	WP_012001188.1	Function unknown
Thioredoxin	WP_012001226.1	Post-translational modification, protein turnover, and chaperones
Hypothetical protein	WP_012001228.1	Function unknown
6,7-dimethyl-8-ribityllumazine synthase	WP_012001235.1	Coenzyme transport and metabolism
Iron ABC transporter ATP-binding protein	WP_012001386.1	Inorganic ion transport and metabolism
Hypothetical protein	WP_012001503.1	Function unknown
3,4-dihydroxy-2-butanone-4-phosphate synthase	WP_012139903.1	Coenzyme transport and metabolism
UDP-glucose 4-epimerase	WP_012140196.1	Cell wall/membrane/envelope biogenesis
Hypothetical protein	WP_012140308.1	Function unknown
Hypothetical protein	WP_012140384.1	Function unknown
Membrane protein	WP_012140437.1	Post-translational modification, protein turnover, and chaperones
ABC transporter substrate-binding protein	WP_012140548.1	Inorganic ion transport and metabolism
Superoxide dismutase	WP_012140576.1	Inorganic ion transport and metabolism
Transcription elongation factor GreA	WP_035142507.1	Transcription
Phosphoribosylformylglycinamidine synthase	WP_048809794.1	Nucleotide transport and metabolism
Hypothetical protein	WP_048809867.1	Function unknown
Endoribonuclease	WP_048809890.1	Translation, ribosomal structure and biogenesis
Protein-export membrane protein SecD	WP_054196430.1	Intracellular trafficking, secretion, and vesicular transport


The normalized total spectral count was used to quantitatively compare protein expression between *C. concisus* 13826 cultured on HBA and HBA^fum0.4^. Of the 355 proteins detected in both conditions, 31 proteins displayed a significant difference in fold change (*p* < 0.05, **Table 6**). Of these, 14 proteins were detected to be significantly less abundant in *C. concisus* when cultured on HBA^fum0.4^ as compared to the same *C. concisus* strain cultured on HBA, and the remaining 17 proteins were significantly more abundant (**Table 6**).

**Table 6:** Proteins with a significant changed of expression profile when cultured on HBA plates and on HBA^fum0.4^.

Protein	Accession number	*t*-Test (*p* < 0.05)	Fold change	Functional category
Flavocytochrome c	WP_012140366.1	0.0002	0.06	Energy production and conversion
Thiosulfate reductase	WP_012140134.1	0.0022	0.07	Energy production and conversion
Dehydrogenase	WP_012140508.1	0.0097	0.2	Energy production and conversion
Trimethylamine-N-oxide reductase	WP_012001034.1	0.0001	0.3	Energy production and conversion
Aryl-sulfate sulfotransferase	WP_012140335.1	0.0059	0.3	Function unknown
L-asparaginase	WP_012140623.1	0.0002	0.4	Amino acid transport and metabolism
Methyl-accepting chemotaxis protein	WP_012001460.1	0.0058	0.4	Cell motility, signal transduction mechanisms
Flavocytochrome c	WP_004317416.1	0.0025	0.5	Energy production and conversion
Peptidase M20	WP_012001703.1	0.0078	0.5	Amino acid transport and metabolism
Biotin attachment protein	WP_012001365.1	0.0380	0.5	Energy production and conversion
Flagellin	WP_012140492.1	0.0250	0.6	Cell motility
Hypothetical protein	WP_012001270.1	0.0160	0.7	Function unknown
DNA-binding protein	WP_012001719.1	0.0250	0.7	Replication, recombination and repair
Long-chain-fatty-acid-CoA ligase	WP_012001496.1	0.0260	0.8	Cell wall/membrane/envelope biogenesis
50S ribosomal protein L2	WP_002941522.1	0.0400	1.4	Translation, ribosomal structure, and biogenesis
30S ribosomal protein S2	WP_021091647.1	0.0015	1.5	Translation, ribosomal structure, and biogenesis
Hydrogenase accessory protein HypB	WP_002941014.1	0.0160	1.5	Transcription, post-translational modification, protein turnover, and chaperones
Hypothetical protein	WP_002941798.1	0.0260	1.5	Function unknown
50S ribosomal protein L15	WP_002941506.1	0.0250	1.6	Translation, ribosomal structure and biogenesis
Phenylalanine-tRNA ligase subunit beta	WP_048809769.1	0.0130	1.7	Translation, ribosomal structure and biogenesis
ABC transporter substrate-binding protein	WP_012001287.1	0.0150	1.7	Carbohydrate transport and metabolism
Glutamyl-tRNA amidotransferase	WP_048809879.1	0.0160	1.7	Translation, ribosomal structure and biogenesis
Peptide ABC transporter substrate-binding protein	WP_012001388.1	0.0072	1.9	Inorganic ion transport and metabolism
3-oxoacyl-[acyl-carrier-protein] reductase	WP_012140278.1	0.0160	1.9	Lipid transport and metabolism
Iron transporter	WP_009294901.1	0.0080	2.3	Inorganic ion transport and metabolism
Succinate dehydrogenase	WP_012001794.1	0.0120	2.5	Energy production and conversion
Heterodisulfide reductase subunit B	WP_012001792.1	0.0430	2.6	Energy production and conversion
Hypothetical protein	WP_012001093.1	0.0130	3.4	Function unknown
Molybdopterin molybdenumtransferase MoeA	WP_002942864.1	0.0160	5.5	Coenzyme transport and metabolism
2-Cys peroxiredoxin	WP_012140581.1	0.0058	7	Post-translational modification, protein turnover, and chaperones
(2Fe-2S)-binding protein	WP_012001793.1	0.0120	14	Energy production and conversion


*The fold change reflects the spectral counts of a protein in *C. concisus* 13826 cultured on HBA plates divided by the spectral counts of the same protein in *C. concisus* 13826 cultured on HBA^*fum0.4*^.*

## Discussion

We investigated the effects of sodium fumarate on the growth of IBD associated bacterial species *C. concisus* and other enteric species. Additionally, proteprotein expression differences when cultured on HBA and HBA^fum0.4^ were also examined in *C. concisus*. We found that sodium fumarate supplementation into culture media alone affected the growth of *C. concisus* and some other enteric bacterial species. In addition, sodium fumarate altered the protein expression in *C. concisus*.

Fumaric acid is a widely used food additive, present in a variety of food products such as candy, jelly, juices, and flat breads. Both fumaric acid and monosodium fumarate are acidic (**Table 2**), which would inhibit the growth of *C. concisus* as shown by our previous study (Ma et al., 2015). However, the consumed fumaric acid will be neutralized in the intestinal tract. As the pH increases, the dissociation of fumaric acid causes a shift to monosodium fumarate and then ultimately to sodium fumarate at neutral pH. The human small intestine has a pH of 6.63 ± 0.53 (Evans et al., 1988); thus, the prevailing fumarate salt is likely to be sodium fumarate. The sodium fumarate in this study therefore represents the neutralized product of fumaric acid and monosodium fumarate in the intestinal tract.

Sodium fumarate at concentrations between 0.05 and 0.4% increased the growth of *C. concisus*. This finding suggests that fumaric acid from dietary sources may enhance the growth of *C. concisus* in the intestinal tract where it is neutralized. Sodium fumarate, although used less frequently as a food additive, may also enhance the growth of *C. concisus* in the gastrointestinal tract. As *C. concisus* was previously shown to be associated with IBD and that virulent strains could damage the intestinal epithelial barrier (Nielsen et al., 2011; Ismail et al., 2012), the data from our study suggests that patients with IBD should consider avoiding excessive consumption of foods containing fumaric acid or its sodium salts. *C. concisus* consists of two genomospecies and each genomospecies contains diverse strains (Chung et al., 2016). In our study, 0.4% sodium fumarate increased the growth of all *C. concisus* strains examined, with differences in the increments dependent on strain (**Table 3**).

At concentrations of 1% sodium fumarate, the growths of some *C. concisus* strains were inhibited, and at a concentration of 2%, the growth of all strains examined was inhibited. However, these concentrations of sodium fumarate are unlikely to be reach in the intestinal tract from dietary source.

Despite the association between *C. concisus* and IBD, the isolation rates of *C. concisus* from enteric samples are low (Mahendran et al., 2011; Mukhopadhya et al., 2011). Our finding that 0.4% sodium fumarate increases the growth of all *C. concisus* strains suggest that it can be used to enhance the isolation of *C. concisus* from enteric samples. We also found that 0.4% sodium fumarate affected *C. concisus* protein expression. Most of the proteins that decreased in expression when *C. concisus* was cultured on HBA^fum0.4^ were involved in metabolism and most of the proteins with increased expression were involved in nutrient acquisition (**Tables 4**, **5**, **6**). These findings indicate that sodium fumarate has increased the growth most likely by affecting energy generation.

We also examined the effects of sodium fumarate on the growth of additional enteric bacterial species including *B. fragilis*, *B. vulgatus*, *E. faecalis*, and *E. coli*. In general, sodium fumarate showed less effect on the growth of these bacterial species as compared to *C. concisus*. For example, sodium fumarate did not affect the growth of *B. fragilis* at all concentrations tested, and a concentration of 0.05% sodium fumarate did not affect the growth of all 4 enteric species. Although sodium fumarate increased the growth of *E. coli* and *E. faecalis* at a concentration of 0.4%, the fold change was only 1.58 and 1.2, respectively (**Figure 5**). These results suggest that sodium fumarate from dietary source is more like to affect the growth of *C. concisus* in the intestinal tract rather than these enteric species.

As this study was performed under laboratory conditions, future studies should be conducted using animal models or in human volunteers to more accurately reflect the effects of food additives on the bacterial species in the gastrointestinal tract. Clinical studies can also be conducted to examine whether the removal of fumaric acid and its salts from the diet of patients with IBD can help improve their condition.

In summary, the results from this study suggest that it may be prudent for patients with IBD to avoid the overconsumption of foods containing fumaric acid or its sodium salts. Additionally, HBA^fum0.4^ plates can be used for the increased probability of isolating *C. concisus* from clinical samples. This was the first study showing that sodium fumarate, the neutralized product of the food additives fumaric acid and monosodium fumarate, affects the growth of IBD associated *C. concisus* in a dose- and strain-dependent manner.

## Ethics Statement

No patient samples were collected in this study and all *C. concisus* strains used in this study were isolated in previous studies. Ethics approval was not required as per institutional and national guidelines.

## Author Contributions

RM, SY, HL, and FL performed the experiments and bioinformatics analyses. MG, SR, and RL provided an important feedback on clinical aspect. LZ and RM conceived the project. RM and LZ played a major role in writing the manuscript. All authors have read the manuscript and provided feedback. All authors have approved the final version of the manuscript.

## Conflict of Interest Statement

The authors declare that the research was conducted in the absence of any commercial or financial relationships that could be construed as a potential conflict of interest.
